# Amyloid β neurotoxicity is IDO1–Kyn–AhR dependent and blocked by IDO1 inhibitor

**DOI:** 10.1038/s41392-020-0188-9

**Published:** 2020-06-12

**Authors:** Zhenzhen Duan, Shengnan Zhang, Heng Liang, Zikang Xing, Leilei Guo, Lei Shi, Lisha Du, Chunxiang Kuang, Osamu Takikawa, Qing Yang

**Affiliations:** 1 State Key Laboratory of Genetic Engineering, School of Life Sciences, Institute of Science and Technology for Brain-Inspired Intelligence, MOE Engineering Research Center of Gene Technology, Shanghai Engineering Research Center of Industrial Microorganisms, Fudan University, Handan Road 220, Shanghai, 200433 China; 2 School of Chemical Science and Engineering, Tongji University, Siping Road 1239, Shanghai, 200092 China; 3 National Institute for Longevity Sciences, National Center for Geriatrics and Gerontology, 35 Gengo, Morioka, Obu, Aichi 474-8511 Japan

**Keywords:** Diseases of the nervous system, Senescence

**Dear Editor,**

Alzheimer’s disease (AD), the most common neurodegenerative disorder and a leading cause of dementia is characterized by progressive memory deficits, cognitive impairment and personality changes. The accumulation of extracellular amyloid beta (Aβ) plaques consisted by Aβ peptide and intracellular neurofibrillary tangles (NFTs) composed by misfolded hyperphosphorylated tau are main pathological hallmarks of AD, which ultimately lead to the dysfunction and loss of synapses and the eventual death of neuron. Neuroinflammation plays a causal role in AD pathogenesis. While the mechanism underlying neuroinflammation-induced AD pathology has not been fully elucidated, increasing evidence proposed that the kynurenine pathway (KP), a major route for l-tryptophan (l-Trp) catabolism, plays an important role.^1^

The KP metabolites are causative of AD pathology. Kynurenine (Kyn), the first stable intermediate in KP, can be converted to two kinds of intermediates of neurotoxic 3-hydroxykynurenine (3-HK), 3-hydroxyanthranilic acid (3-HAA) and quinolinic acid (QUIN). and neuroprotective kynurenic acid (KYNA). In AD cases, the aberrant levels of QUIN, 3-HK, and KYNA are commonly observed.^1^ More importantly, activation of the Trp-degrading indoleamine 2,3-dioxygenase 1 (IDO1), the rate-limiting enzyme of KP, is a pathogenic factor of Aβ-related inflammation in AD.^1^ In addition, the increased expression of IDO1 and the accumulation of QUIN co-localized with the well-known AD hallmarks, Aβ deposits and Tau have been observed in AD brain.^2^ Elevated levels of IDO1 and QUIN have been mainly observed in microglia and astrocytes which are the mediators of neuroinflammation in AD^1^, known sites of KP catabolism. While in situ data on the KP in neurons are limited. The effect of Aβ oligomers on IDO1 and KP in neurons still remains unclear.

Furthermore, Kyn has been recognized as an endogenous agonist of aryl hydrocarbon receptor (AhR).^2^ AhR activation by Kyn has a deleterious role in cerebral ischemia and Kyn-AhR pathway is identified as a potential therapeutic target for stroke. The physiological and pathological roles of AhR in central nervous system are still unclear yet, least of AD. However, it was found that there is a crosstalk between AhR and Wnt/β-catenin signal pathway.^3^ Down-regulation of Wnt/β-catenin signaling pathway increases Tau phosphorylation, induces neuronal cell death and can be involved in the cognitive decline associated with aging and the physiopathology of AD.^4^ However, the impact of AhR on Wnt/β-catenin function in AD is largely unknown.

First, using SD rat primary hippocampal neurons and mouse hippocampal neuronal HT22 cells, we explored the effects of Aβ on IDO1–Kyn–AhR and Wnt/β-catenin signaling pathways and the reversal effects of IDO1 inhibitors (RY101, RY103, or 1-L-MT) on Aβ-induced neuronal damage. It was shown that different concentrations of Aβ led to a dose-dependent increase in the expression of IDO1 and AhR, β-catenin phosphorylation (phosphorylated β-catenin level/β-catenin level) and Tau phosphorylation, but decrease in GSK3β phosphorylation (phosphorylated GSK3β level/GSK3β level) (Supplementary Fig. S1a). IDO1 inhibitors decreased the expressions of IDO1 and AhR, increased GSK3β phosphorylation, and decreased β-catenin phosphorylation and Tau phosphorylation (Fig. 1a and Supplementary Fig. S1c, d). The mRNA level of CYP1A1, the target gene of AhR, was increased by Aβ treatment, while IDO1 inhibitors reversed the effect of Aβ (Fig. 1b and Supplementary Fig. S1e). Supporting the above data, the immunofluorescence staining showed that Aβ increased the expressions of IDO1 and AhR, which were reversed by IDO1 inhibitors (Supplementary Fig. S1b, f). IDO1 inhibitors could attenuate Aβ-induced injury on neuron skeleton by restoring the expression of neuronal-specific cytoskeletal protein class III β-tubulin (Supplementary Fig. S1b). Aβ increased the expression of AhR both in cytoplasm and nuclei, IDO1 inhibitor could reverse the increased AhR expression in the cytoplasm but not in the nuclei (Supplementary Fig. S1g). Similarly, Kyn increased the expression of AhR in both cytoplasm and nucleus, and increased the CYP1A1 mRNA level (Supplementary Fig. S1h, i). Aβ treatment significantly induced neuron apoptosis and decreased the immunostaining density of postsynaptic density protein 95 (PSD95), which were relieved by the supplement of IDO1 inhibitors (Fig. 1c, d and Supplementary Fig. S2a, c, h, i). Aβ impaired primary hippocampal neuron by damaging synapse and breaking neuron connections (Supplementary Fig. S2b). IDO1 inhibitors reversed the effects of Aβ on the neurite extension (Supplementary Fig. S2d, e) and the viability of HT22 cells (Supplementary Fig. S2f, g).

**Fig. 1:**
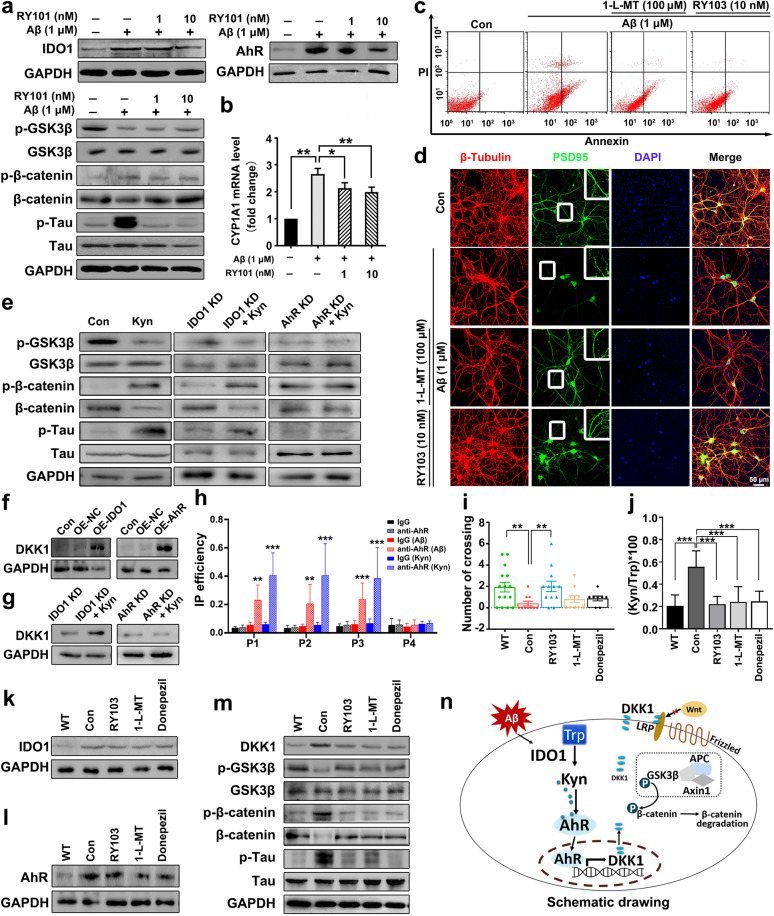
Amyloid β neurotoxicity is IDO1-Kyn-AhR dependent and blocked by IDO1 inhibitor. **a**–**d** SD rat primary hippocampal neurons were treated with Aβ (1 μM) or Aβ (1 μM) plus RY101 (1 or 10 nM), 1-L-MT (100 μM), or RY103 (10 nM) for 24 h. **a** Expressions of IDO1-Kyn-AhR, Wnt/β-catenin signaling pathway proteins, and p-Tau determined by western blot. **b** mRNA expression of CYP1A1 quantified by qPCR. **c** Apoptosis of neurons evaluated by flow cytometry. Annexin V-FITC is used for cytomembrane staining and PI is used for nucleus staining. **d** Immunostaining of postsynaptic marker PSD95 (green), neuronal nuclei (DAPI, blue), and β-tubulin (red) (×400 magnification, scale bar = 50 μm). The upright panel exhibited an enlarged view of the representative region in the white box. **e** The effect of Kyn (200 μM, 24 h) on the expressions of Wnt/β-catenin signaling pathway proteins and p-Tau in wild-type, IDO1 stable knockdown (IDO1 KD), and AhR stable knockdown (AhR KD) HT22 cells determined by western blot. **f** Expressions of DKK1 in IDO1 stable overexpressing (IDO1 OE) or AhR stable overexpressing (AhR OE) HT22 cells determined by western blot. Con group represents wild-type HT22 cells without treatment, OE-NC group represents wild-type HT22 cells transfected with empty vector. **g** The effect of Kyn (200 μM, 24 h) on DKK1 expression in IDO1 stable knockdown (IDO1 KD) or AhR stable knockdown (AhR KD) HT22 cells determined by western blot. **h** ChIP analysis of AhR binding to DKK1 promoter in HT22 cells treated with Kyn (200 μM, 24 h) or Aβ (5 μM, 24 h). ChIP assay was performed with control IgG or anti-AhR antibodies. Immunoprecipitated DNA was examined using qPCR and primers specific for the DKK1 promoter. **i** Morris water maze (MWM) test for the number of platform location crossings. (*n* = 8–15 mice in each group). **j** Concentrations of Trp and Kyn in serum were determined by HPLC and Kyn/Trp ratio was calculated (*n* = 5–9 mice in each group). **k**–**m** Expressions of IDO1-Kyn-AhR, DKK1, and Wnt/β-catenin signaling pathway proteins in the hippocampus determined by western blot. (*n* ≥ 3 mice in each group). **n** Schematic of Aβ neurotoxicity regulates Wnt/β-catenin signaling pathway via IDO1-Kyn-AhR pathway. Results shown in **a**–**h** are representative of at least three independent experiments. Results shown in **i**–**m**, *n* ≥ 3 mice in each group. The data of **e** and **g** were analyzed by Student’s *t* test, the other data were analyzed by one-way ANOVA followed by Dunnett’s post hoc test. The data of **i** and **j** were expressed as the mean ± SD. The data of **b** and **h** were expressed as the mean ± SEM. *p < 0.05, **p < 0.01, ***p < 0.001. The mRNA values are normalized to the level of actin mRNA

Then, we investigated the regulation of IDO1–Kyn–AhR on Wnt/β-catenin signaling pathway and Tau phosphorylation in HT22 cells. The stable IDO1 over-expressing (IDO1 OE), AhR over-expressing (AhR OE), IDO1 knockdown (IDO1 KD), and AhR knockdown (AhR KD) HT22 cell lines were constructed. As shown in Fig. S3a, b, GSK3β phosphorylation was decreased while β-catenin phosphorylation and Tau phosphorylation were increased in IDO1 or AhR OE HT22 cells, suggesting that IDO1 or AhR over-expression down-regulated Wnt/β-catenin signaling. Additionally, it was found that Kyn down-regulated Wnt/β-catenin signaling and increased Tau phosphorylation in wild type HT22 cell lines (Fig. 1e). In IDO1 KD HT22 cells, the addition of Kyn still down-regulated Wnt/β-catenin signaling and increased Tau phosphorylation (Fig. 1e). However, in AhR KD HT22 cells, the addition of Kyn did not affect Wnt/β-catenin signaling and Tau phosphorylation (Fig. 1e). Using HT22 cells transiently transfected with siRNA targeting IDO1 (siIDO1), AhR (siAhR), or nontargeting siRNA (NC), the effects of Aβ on the expressions of IDO1, AhR, CYP1A1, and Wnt/β-catenin signaling pathway proteins were examined. This result indicated that IDO1 and AhR were involved in the Aβ-induced Wnt/β-catenin signaling pathway down-regulation and Tau phosphorylation in HT22 cells (Supplementary Fig. S3c–f).

Further, we sought to clarify that in neurons Aβ down-regulated Wnt/β-catenin signaling pathway through the ability of Aβ to induce Dickkopf-1 (DKK1) via IDO1–Kyn–AhR pathway. DKK1, a negative modulator of Wnt/β-catenin signaling pathway, involves in Aβ-related neuron damage^4^ and is suggested to be regulated by AhR. ChIP analysis using crosslinked chromatin from the HT22 cells defined that AhR could bind to the dioxin-responsive elements (DRE) sites of DKK1 promoter (Fig. 1h and Supplementary Fig. S4a). The expression of DKK1 was significantly increased in IDO1 OE or AhR OE HT22 cells (Fig. 1f). Also, DKK1 expression in the hippocampus of APOE^−/−^ and APP/PS1 mice was higher than that of WT mice (Supplementary Fig. S4b). Kyn increased DKK1 expression in IDO1 KD HT22 cells but not in AhR KD HT22 cells (Fig. 1g). The expression of DKK1 in HT22 cells was increased upon the Kyn or Aβ treatment (Supplementary Fig. S4c, d). While 1-L-MT reversed the effect of Aβ on DKK1 expression (Supplementary Fig. S4e). The expression of DKK1 in IDO1-deficient or AhR-deficient HT22 cells supplemented with Aβ was lower than that in NC group treated with Aβ (Supplementary Fig. S4f, g). These results indicated that the modulation of IDO1–Kyn–AhR pathway on Wnt/β-catenin signaling pathway was dependent on DKK1.

At last, we explored the effects of IDO1 inhibitor on cognitive performance of APP/PS1 mice. RY103 was found to be able to penetrate the blood–brain barrier (BBB) in vivo (Supplementary Fig. S5a). Morris water maze (MWM) test for escape latency (Supplementary Fig. S5b), time spent in the target quadrant (Supplementary Fig. S5c), distance spent in the target quadrant (Supplementary Fig. S5d) and the number of platform location crossings (Fig. 1i) showed that RY103 improved the cognitive function of APP/PS1 mice. IDO1 inhibitors decreased the serum IDO1 activity (Table 1). Furthermore, the expressions of IDO1 and AhR and the mRNA level of CYP1A1 in the hippocampus were all decreased by the administration of IDO1 inhibitors (Fig. 1k, l and Supplementary Fig. S5e). DKK1 expression was found decreased in IDO1 inhibitor groups (Fig. 1m), consequently, Wnt/β-catenin signaling pathway was up-regulated in IDO1 inhibitor groups (Fig. 1m). These data showed that IDO1 inhibitors attenuated the aberrant IDO1–Kyn–AhR and Wnt/β-catenin signaling pathways and exhibited neuroprotective effect in APP/PS1 mice. Our study is one of few studies that focus on studying IDO1 inhibitors on treating AD.^5^

Taken together, for the first time, we demonstrate that in neurons Aβ up-regulated IDO1–Kyn–AhR signal pathway accompanied by the down-regulation of Wnt/β-catenin signaling pathway, which could be reversed by IDO1 inhibitor. We prove that Aβ neurotoxicity is IDO1–Kyn–AhR dependent. Activation of IDO1–Kyn–AhR, through DKK1, down-regulated Wnt/β-catenin pathway to drive Tau pathology and neurotoxicity and can be blocked by IDO1 inhibitors. Our data suggest the importance of aberrant IDO1–Kyn–AhR signaling in AD neuropathology and shed new light on the use of IDO1 inhibitor in the treatment of the disease.

## Supplementary information

Supplementary Materials
