# Supplementary material for: Amyloid β neurotoxicity is IDO1–Kyn–AhR dependent and blocked by IDO1 inhibitor
Source: Signal Transduct Target Ther. 2020 Jun 12;5:96. doi: 10.1038/s41392-020-0188-9 (PMC7293319; doi:10.1038/s41392-020-0188-9)
Supplement: Supplementary file 1 — Supplementary Materials [file 41392_2020_188_MOESM1_ESM.docx]

**Supplemental Materials**

**Amyloid β neurotoxicity is IDO1-Kyn-AhR dependent and blocked by IDO1 inhibitor**

Zhenzhen Duan^a^, Shengnan Zhang^a^, Heng Liang^a^, Zikang Xing^a^, Leilei Guo^a^, Lei Shi^a^, Lisha Du^a^, Chunxiang Kuang^b^, Osamu Takikawa^c^, Qing Yang^a*^

^a^State Key Laboratory of Genetic Engineering, School of Life Sciences, Institute of Science and Technology for Brain-Inspired Intelligence, MOE Engineering Research Center of Gene Technology, Shanghai Engineering Research Center of Industrial Microorganisms, Fudan University, Handan Road 220, Shanghai, 200433, China

^b^School of Chemical Science and Engineering, Tongji University, Siping Road 1239, Shanghai, 200092, China

^c^National Institute for Longevity Sciences, National Center for Geriatrics and Gerontology 35 Gengo, Morioka, Obu, Aichi 474-8511, Japan

*Corresponding author:

Qing Yang, address: State Key Laboratory of Genetic Engineering, School of Life Sciences, Institute of Science and Technology for Brain-Inspired Intelligence, MOE Engineering Research Center of Gene Technology, Shanghai Engineering Research Center of Industrial Microorganisms, Fudan University, Songhu Road 2005, Shanghai, China, telephone & fax number: +86-021-31246641, E-mail: yangqing68@fudan.edu.cn

*Keywords*: Indoleamine 2,3-dioxygenase 1; Kynurenine; Aryl hydrocarbon receptor; IDO1-Kyn-AhR signaling pathway; Indoleamine 2,3-dioxygenase 1 inhibitor; Alzheimer’s disease; Wnt/β-catenin signaling pathway

**This file includes:**

Figure legend for Fig. 1. Amyloid β neurotoxicity is IDO1-Kyn-AhR dependent and blocked by IDO1 inhibitor 3

Supplemental Figures 5

Fig. S1. Aβ up-regulates IDO1-Kyn-AhR and down-regulates Wnt/β-catenin signaling pathway in neurons 5

Fig. S2. IDO1 inhibitors reverse Aβ-induced apoptosis and Aβ-impaired neuroplasticity in neurons 8

Fig. S3. Aβ inhibits Wnt/β-catenin signaling by up-regulating IDO1-Kyn-AhR pathway in HT22 cells 11

Fig. S4. IDO1-Kyn-AhR pathway modulates DKK1, the negative modulator of Wnt/β-catenin signaling pathway 13

Fig. S5. IDO1 inhibitor improves the cognitive performance of APP/PS1 mice 15

Tables 17

Methods and materials 20

References 27

**Figure 1 (Fig. 1). Amyloid β neurotoxicity is IDO1-Kyn-AhR dependent and blocked by IDO1 inhibitor.** a-d. SD rat primary hippocampal neurons were treated with Aβ (1 μM) or Aβ (1 μM) plus RY101 (1 or 10 nM), 1-L-MT (100 μM) or RY103 (10 nM) for 24 h. a. Expressions of IDO1-Kyn-AhR, Wnt/β-catenin signaling pathway proteins and p-Tau determined by western blot. b. mRNA expression of CYP1A1 quantified by qPCR. c. Apoptosis of neurons evaluated by flow cytometry. Annexin V-FITC is used for cytomembrane staining and PI is used for nucleus staining. d. Immunostaining of postsynaptic marker PSD95 (green), neuronal nuclei (DAPI, blue) and β-tubulin (red) (×400 magnification, scale bar = 50 μm). The upright panel exhibited an enlarged view of the representative region in the white box. e. The effect of Kyn (200 µM, 24 h) on the expressions of Wnt/β-catenin signaling pathway proteins and p-Tau in wild type, IDO1 stable knockdown (IDO1 KD) and AhR stable knockdown (AhR KD) HT22 cells determined by western blot. f. Expressions of DKK1 in IDO1 stable over-expressing (IDO1 OE) or AhR stable over-expressing (AhR OE) HT22 cells determined by western blot. Con group represents wild type HT22 cells without treatment, OE-NC group represents wild type HT22 cells transfected with empty vector. g. The effect of Kyn (200 µM, 24 h) on DKK1 expression in IDO1 stable knockdown (IDO1 KD) or AhR stable knockdown (AhR KD) HT22 cells determined by western blot. h. ChIP analysis of AhR binding to DKK1 promoter in HT22 cells treated with Kyn (200 µM, 24 h) or Aβ (5 µM, 24 h). ChIP assay was performed with control IgG or anti-AhR antibodies. Immunoprecipitated DNA was examined using qPCR and primers specific for the DKK1 promoter. i. Morris water maze (MWM) test for the number of platform location crossings. (n = 8-15 mice in each group). j. Concentrations of Trp and Kyn in serum were determined by HPLC and Kyn/Trp ratio was calculated (n = 5-9 mice in each group). k-m. Expressions of IDO1-Kyn-AhR, DKK1 and Wnt/β-catenin signaling pathway proteins in the hippocampus determined by western blot. (n ≥ 3 mice in each group). n. Schematic of Aβ neurotoxicity regulates Wnt/β-catenin signaling pathway via IDO1-Kyn-AhR pathway. Results shown in a-h are representative of at least three independent experiments. Results shown in i-m, n ≥ 3 mice in each group. The data of Fig. 1e&g were analyzed by Student’s t-test, the other data were analyzed by one-way ANOVA followed by Dunnett’s post hoc test. The data of Fig. 1i&j were expressed as the mean ± SD. The data of Fig. 1b&h were expressed as the mean ± SEM. **p* <0.05, ***p* <0.01, ****p* <0.001. The mRNA values are normalized to the level of actin mRNA.


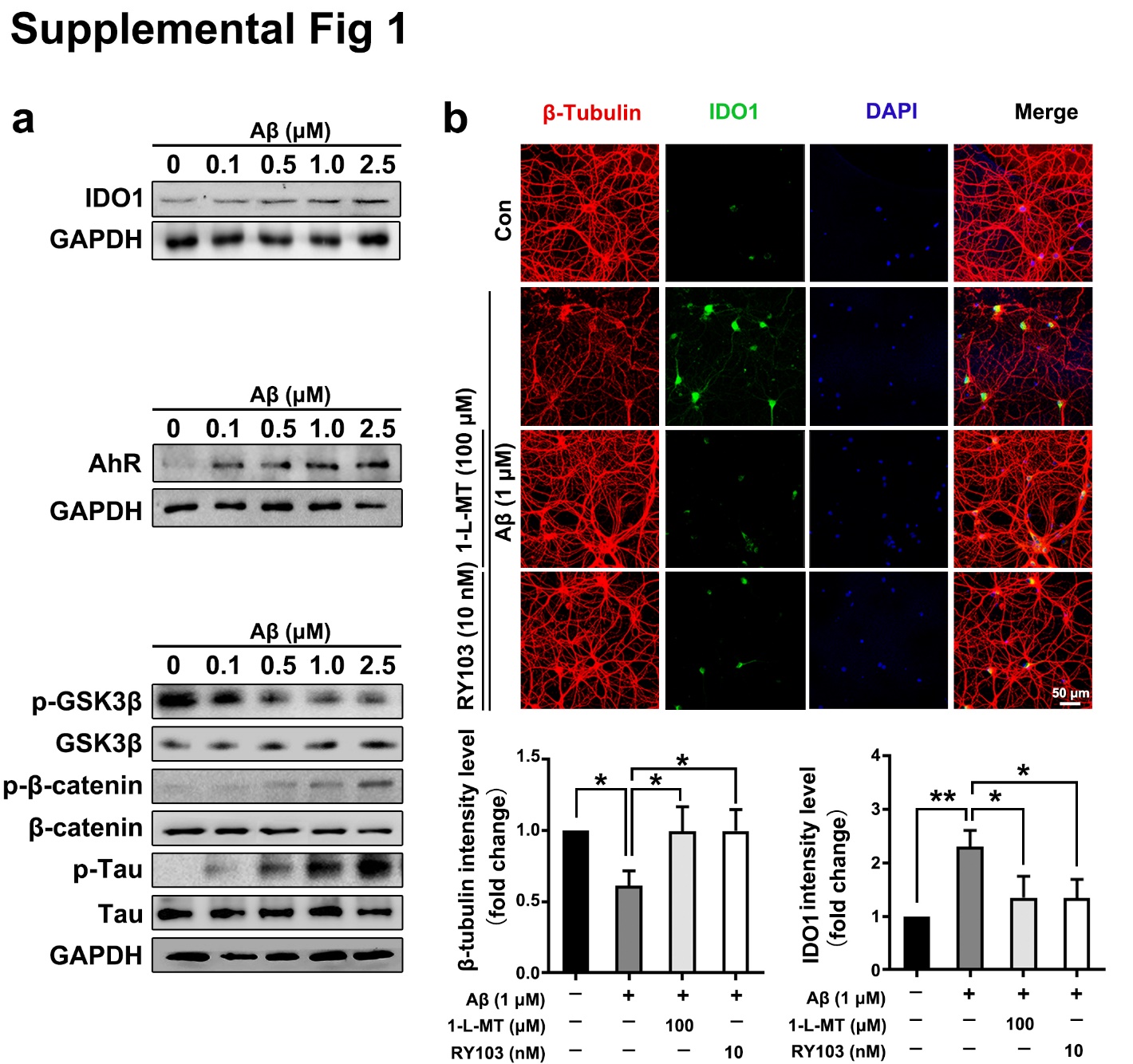


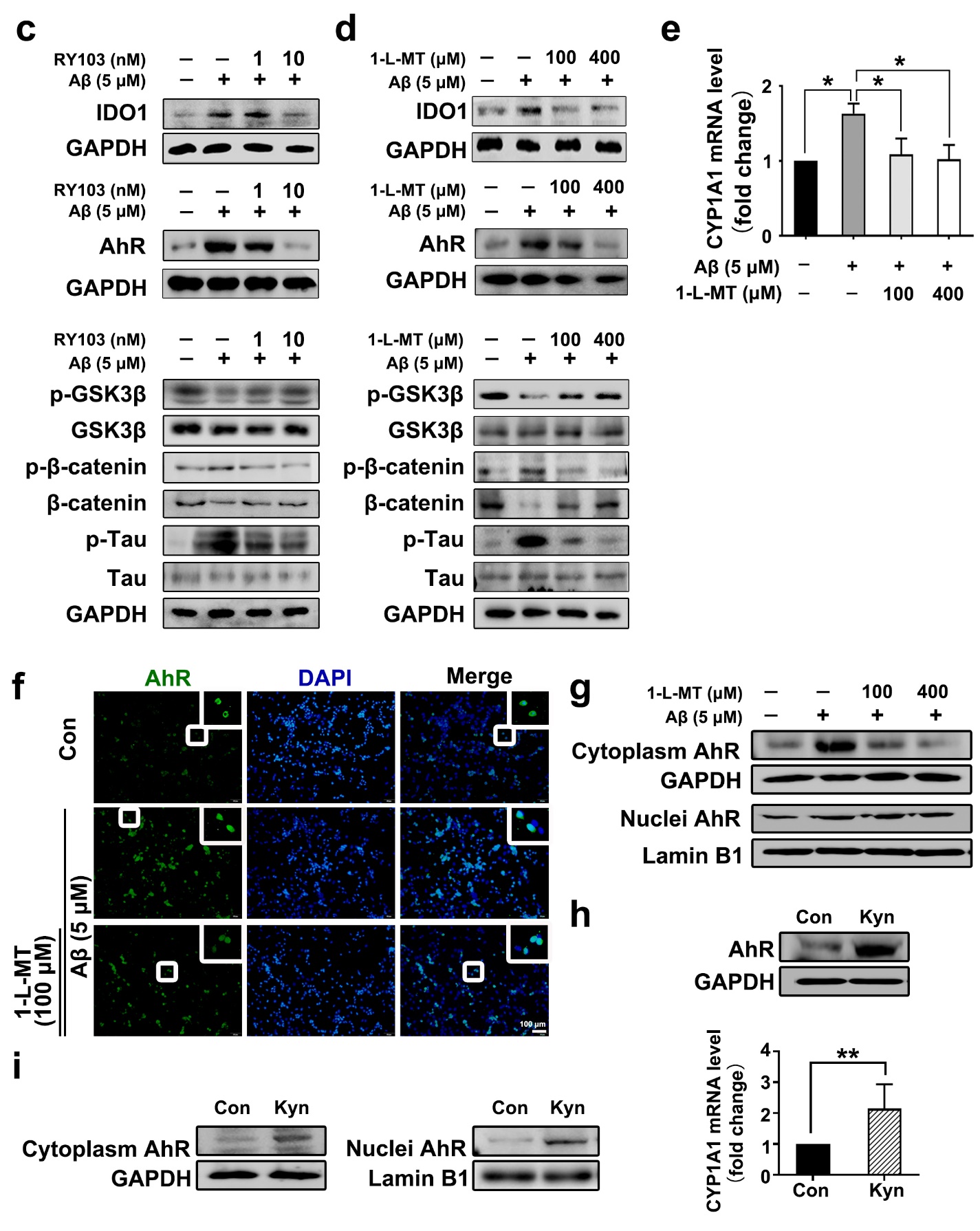


**Supplemental Fig 1 (Fig. S1). Aβ up-regulates IDO1-Kyn-AhR and down-regulates Wnt/β-catenin signaling pathway in neurons while IDO1 inhibitors reverse the effects of Aβ.** a&b. SD rat primary hippocampal neurons were treated with Aβ of different concentrations (0.1, 0.5, 1.0, 2.5 μM) or Aβ (1 μM) plus 1-L-MT (100 μM), RY103 (10 nM) for 24 h. a. Expressions of IDO1-Kyn-AhR, Wnt/β-catenin signaling pathway proteins and p-Tau determined by western blot. b. Immunostaining of IDO1 (green), neuronal nuclei (DAPI, blue) and β-tubulin (red) (×400 magnification, scale bar = 50 μm). c-g. HT22 cells were incubated with Aβ (5 μM), Aβ (5 μM) supplemented with 1-L-MT (100, 400 μM) or RY103 (1, 10 nM) for 24 h. c&d. Expressions of IDO1-Kyn-AhR, Wnt/β-catenin signaling pathway proteins and p-Tau determined by western blot. e. mRNA expression of CYP1A1 quantified by qPCR. f. Immunostaining of AhR (green) and neuronal nuclei (DAPI, blue) (×200 magnification, scale bar = 100 μm). The upright panel exhibited an enlarged view of the representative region in the white box. g. The expression of AhR in the cytoplasm and the nucleus determined by western blot. h&i. HT22 cells were incubated with Kyn (200 μM) for 24 h. h. The AhR expression and mRNA level of CYP1A1. i. The expression of AhR in the cytoplasm and the nucleus determined by western blot. Results are representative of at least three independent experiments. The data of Fig. S1h&i were analyzed by Student’s t-test, the other data were analyzed by one-way ANOVA followed by Dunnett’s post hoc test. All the data were expressed as the mean ± SEM. **p* <0.05, ***p* <0.01. The mRNA values are normalized to the level of actin mRNA.


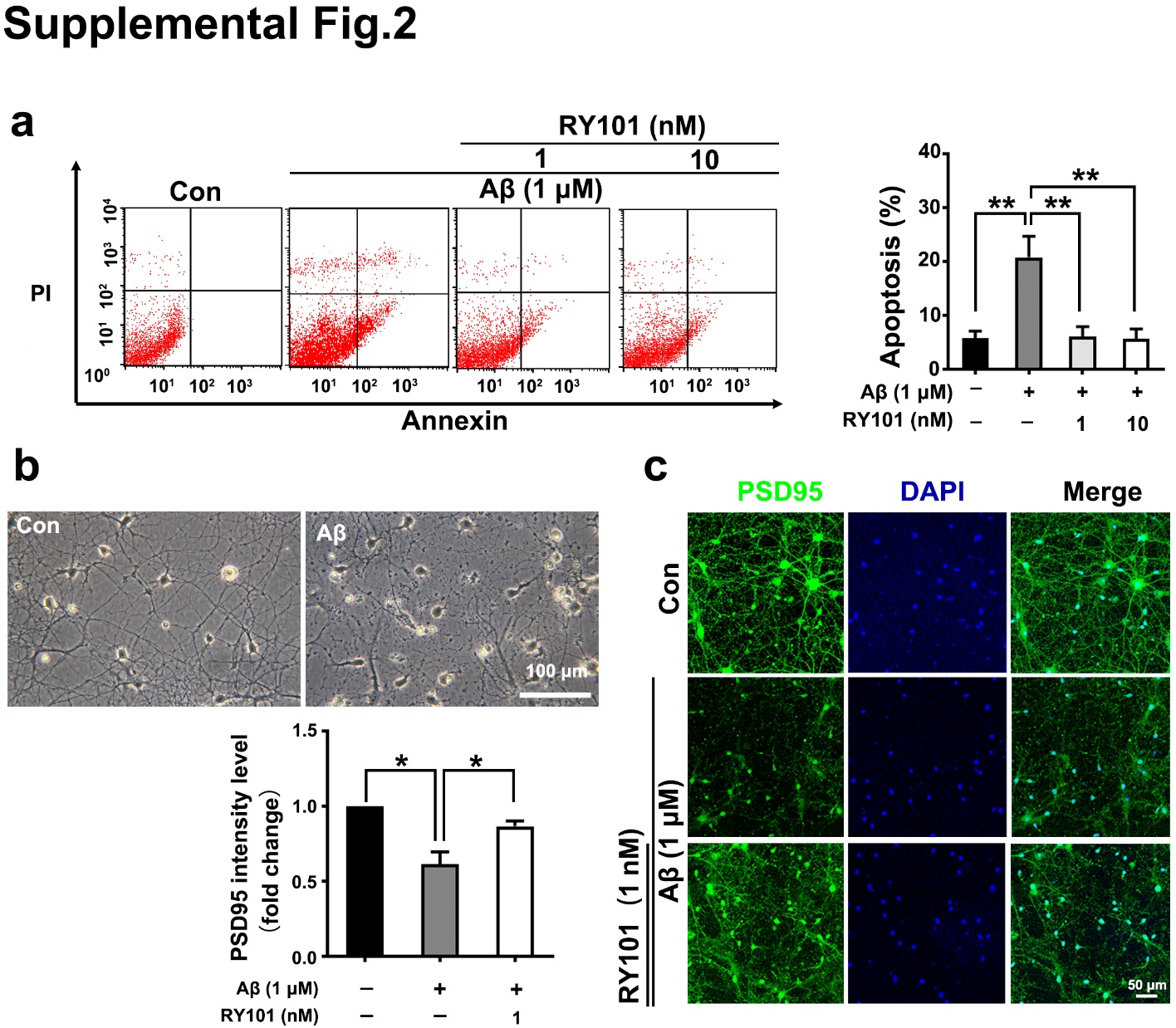


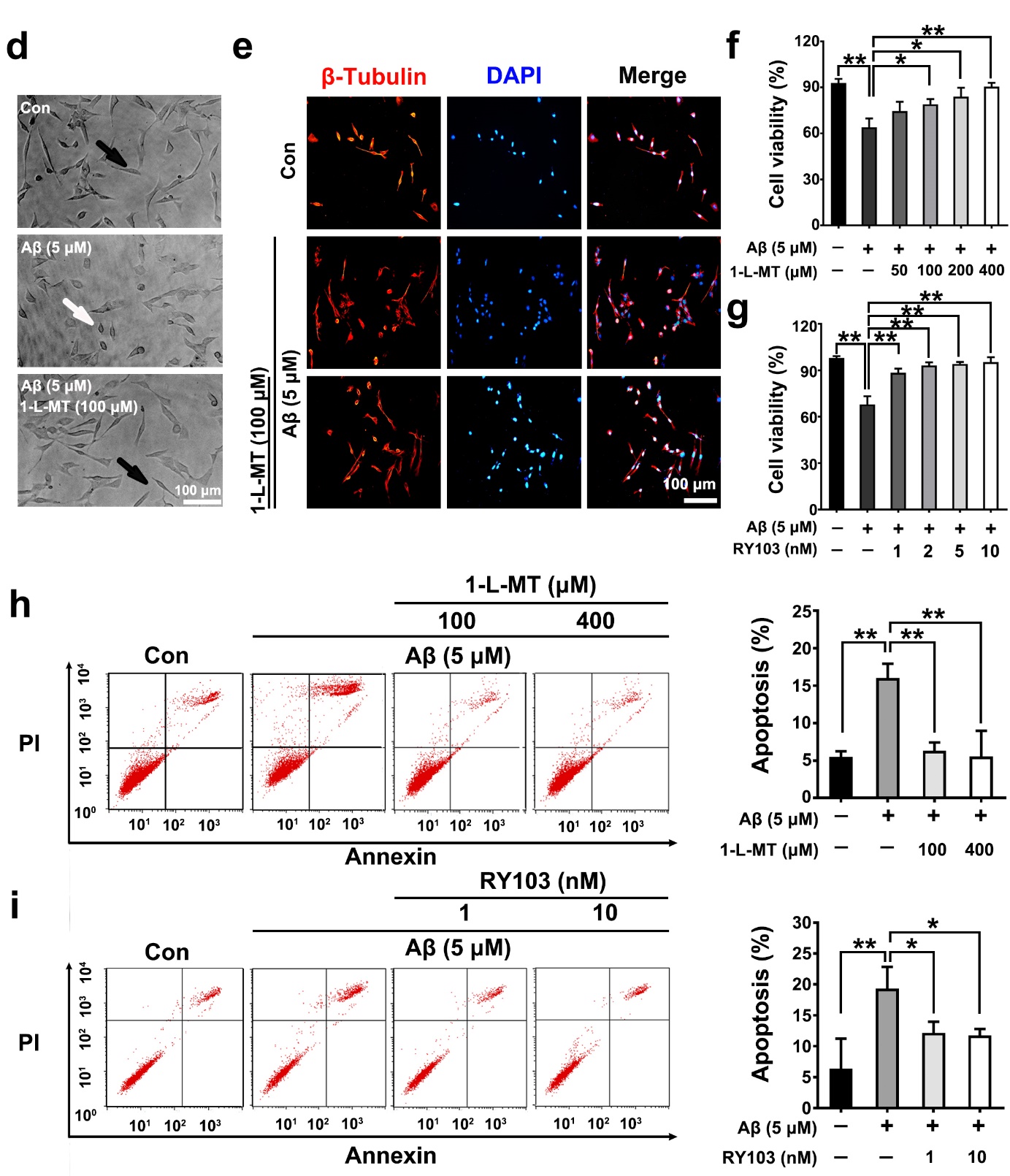


**Supplemental Fig 2 (Fig. S2). Protective effects of IDO1 inhibitors on Aβ-induced damage to neurons.** a-c. SD rat primary hippocampal neurons were treated with Aβ (1 μM) or Aβ (1 μM) plus RY101 (1 or 10 nM) for 24 h. a. Apoptosis of neurons evaluated by flow cytometry. Annexin V-FITC is used for cytomembrane staining and PI is used for nucleus staining. b. Morphological changes of primary neuron due to Aβ treatment recorded by phase-contrast microscopy (×200 magnification, scale bar = 100 μm). c. Immunostaining of postsynaptic marker PSD95 (green) and neuronal nuclei (DAPI, blue) (×400 magnification, scale bar = 50 μm). d-i. HT22 cells were incubated with Aβ (5 μM), Aβ (5 μM) supplemented with 1-L-MT or RY103 for 24 h. d. HT22 cell morphological changes described by phase-contrast microscopy (×200 magnification, scale bar = 100 μm). Black narrows showed long and narrow cells, and white narrows showed shrunk cells. e. Immunostaining of β-tubulin (red) and neuronal nuclei (DAPI, blue) (×200 magnification, scale bar = 100 μm). f&g. Viability of HT22 cells evaluated by Trypan Blue staining. h&i. The apoptosis of HT22 cells evaluated by flow cytometry. Annexin V-FITC is used for cytomembrane staining and PI is used for nucleus staining. Results are representative of at least three independent experiments. All the data were analyzed by one-way ANOVA followed by Dunnett’s post hoc test and expressed as the mean ± SEM. **p* <0.05, ***p* <0.01.


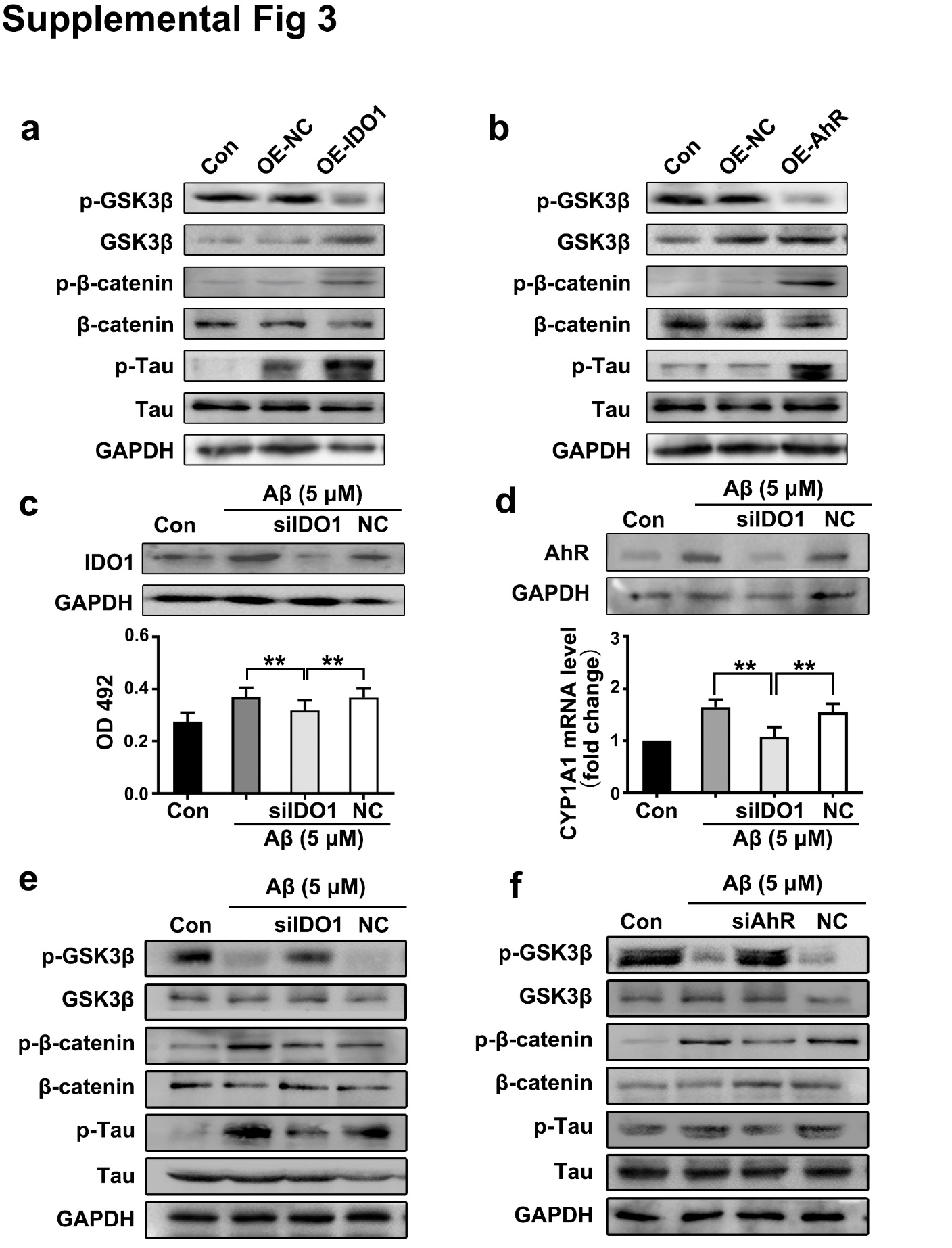


**Supplemental Fig 3 (Fig. S3). Regulation of IDO1-Kyn-AhR on Wnt/β-catenin signaling pathway in HT22 cells.** Con group represents wild type HT22 cells without treatment, OE-NC group represents wild type HT22 cells transfected with empty vector, and NC group represents wild type HT22 cells transfected with nontargeting siRNA. a&b. The expressions of Wnt/β-catenin signaling pathway proteins and p-Tau in IDO1 stable over-expressing (IDO1 OE) and AhR stable over-expressing (AhR OE) HT22 cells determined by western blot. c. Expression and activity of IDO1 in IDO1 deficient (siIDO1) HT22 cells treated with Aβ. d&e. Expressions of AhR, CYP1A1 and Wnt/β-catenin signaling pathway proteins in IDO1 deficient (siIDO1) HT22 cells treated with Aβ. f. Expression of Wnt/β-catenin signaling pathway proteins in AhR deficient (siAhR) HT22 cells treated with Aβ. Results are representative of at least three independent experiments. All the data were analyzed by one-way ANOVA followed by Dunnett’s post hoc test and expressed as the mean ± SEM. ***p* <0.01. The mRNA values are normalized to the level of actin mRNA.


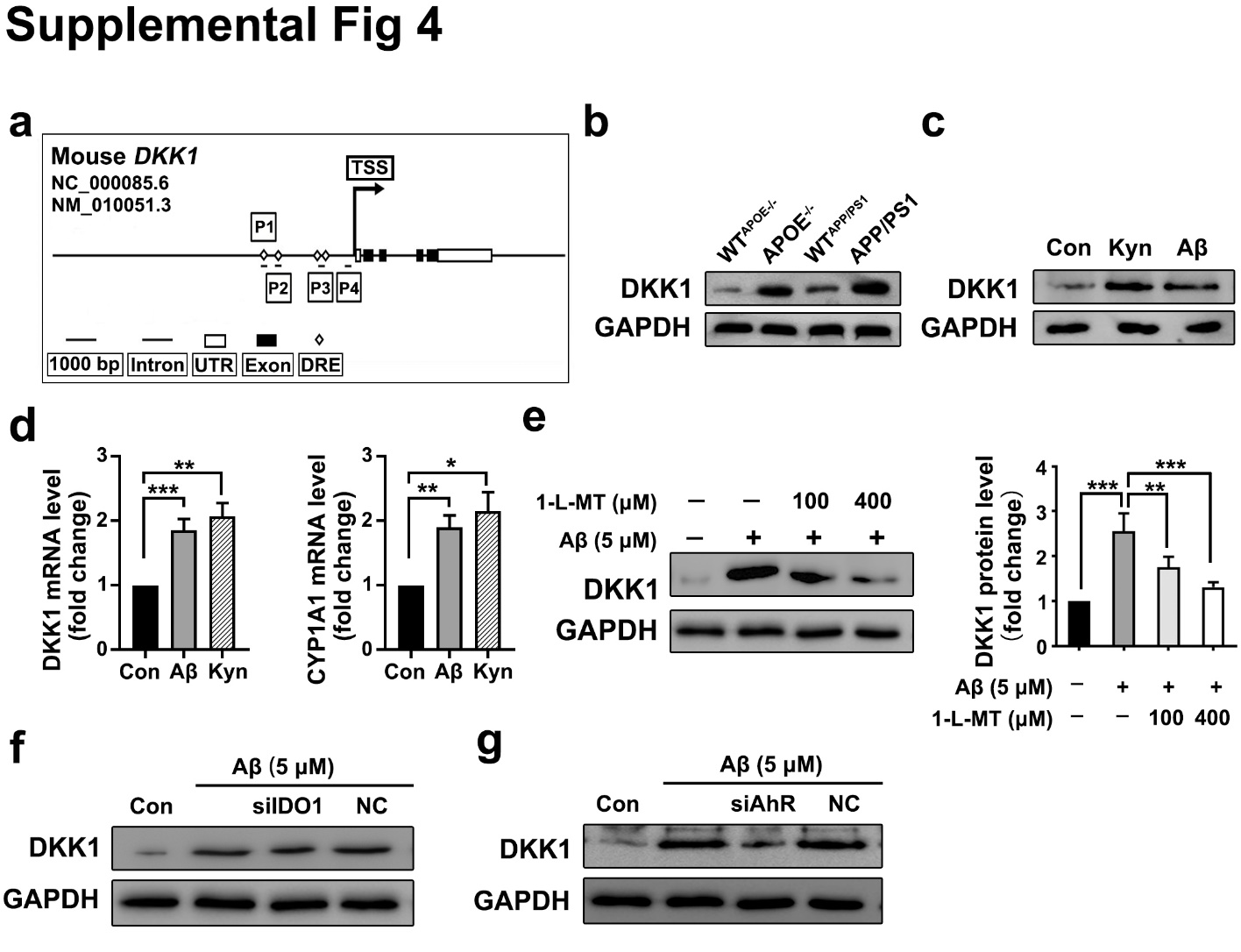


**Supplemental Fig 4 (Fig. S4). IDO1 derived Kyn activates the binding of AhR to DKK1 promoter and up-regulates the expression of DKK1.** a. Schematic of predicted AhR binding sites (DREs) in mouse DKK1 promoter. b. Expression of DKK1 in the hippocampus of APOE^-/-^ mice (3-month-old, male) and APP/PS1 mice (6-month-old, male) determined by western blot. (n = 3 mice in each group). c&d. The effect of Kyn (200 µM, 24 h) or Aβ (5 µM, 24 h) on the protein and mRNA levels of DKK1 in HT22 cells determined by western blot and qPCR. e. Western blot protein detection and quantitation of DKK1 in HT22 cells treated with Aβ or Aβ plus 1-L-MT. f&g. Expressions of DKK1 in IDO1 deficient (siIDO1) or AhR deficient (siAhR) HT22 cells treated with Aβ determined by western blot. Con group represents wild type HT22 cells without treatment, and NC group represents wild type HT22 cells transfected with nontargeting siRNA. Results shown in c-g are representative of at least three independent experiments. Results shown in b, n = 3 mice in each group. The data of Fig. S4b were analyzed by Student’s t-test, the other data were analyzed by one-way ANOVA followed by Dunnett’s post hoc test. All the data were expressed as the mean ± SEM. **p* <0.05, ***p* <0.01, ****p* <0.001. The mRNA values are normalized to the level of actin mRNA.


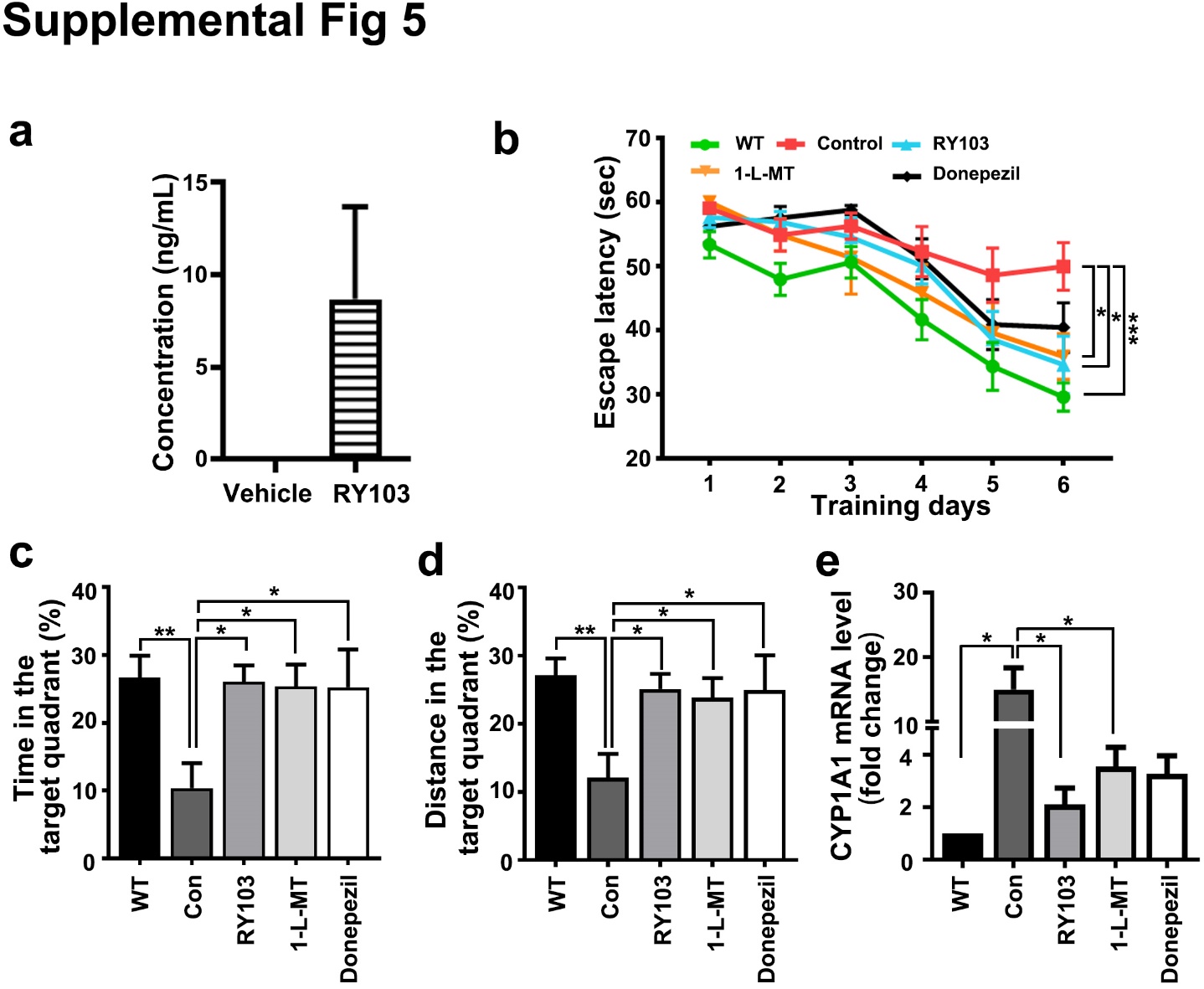


**Supplemental Fig 5 (Fig. S5). IDO1 inhibitors improve the cognitive function of APP/PS1 mice, attenuate the aberrant IDO1-Kyn-AhR and Wnt/β-catenin signaling pathways, and exhibit neuroprotective effect in APP/PS1 mice**. a. The concentration of RY103 in the brain of SD rats 2 h post single oral administration detected by LC-MS. (n = 3 mice in each group). b-d. MWM test for escape latency (b), time spent in the target quadrant (c), distance spent in the target quadrant (d) (n = 8-15 mice in each group). e. mRNA expression of CYP1A1 in the hippocampus quantified by qPCR. (n ≥ 3 mice in each group). The data of escape latency on day 6 were statistically analyzed using a one-way ANOVA (Fig. S5b). The data of Fig. S5c&d were analyzed by one-way ANOVA followed by Dunnett’s post hoc test. The data of Fig. S5a-d were expressed as the mean ± SD. The data of Fig. S5e were expressed as the mean ± SEM. **p* <0.05, ***p* <0.01, ****p* <0.001. The mRNA values are normalized to the level of actin mRNA.

**Table 1.** Concentrations (mean ± SD) of Trp and Kyn and the Kyn/Trp ratio in the serum of APP/PS1 mice administrated with IDO1 inhibitors. n = 5-9 mice in each group.

|  | WT (n=8) |  | APP/PS1 | | | |
| --- | --- | --- | --- | --- | --- | --- |
|  |  |  | Control  (n=6) | RY103  (n=9) | 1-L-MT  (n=6) | Donepezil  (n=5) |
| Trp (μmol/L) | 74.3 ± 20.6 |  | 70.0 ± 10.7 | 67.6 ± 10.2 | 68.2 ±11.2 | 70.3 ± 10.7 |
| Kyn (μmol/L) | 0.14 ± 0.05^***^ |  | 0.38 ± 0.08 | 0.14 ± 0.04^***^ | 0.16 ± 0.06^***^ | 0.18 ± 0.09^***^ |
| (Kyn/Trp) × 100 | 0.19 ± 0.09^***^ |  | 0.54 ± 0.14 | 0.21 ± 0.07^***^ | 0.24 ± 0.21^***^ | 0.26 ± 0.09^***^ |

8-month-old male WT and APP/PS1 mice were administrated with vehicle, IDO1 inhibitors or donepezil for one month. The data were analyzed by one-way ANOVA followed by Dunnett’s post hoc test and expressed as the mean ± SD, ****p* <0.001.

**Table 2.** The sequences of primers used for qPCR analysis of gene expression.

| **Species** | **Genes** | **Forward (5’-3’)** | **Reverse (5’-3’)** |
| --- | --- | --- | --- |
| Mouse | CYP1A1 | CACTAATGGCAAGAGCATGAC | TCACCTTCTGAAGTTTGCTGAC |
|  | DKK1 | CCTACCCTTGCGCTGAAGAT | TGGTCAGAGGGCATGCATATT |
|  | actin | CTGTCCCTGTATGCCTCTG | ATGTCACGCACGATTTCC |
| Rat | CYP1A1 | CGCTAATGGCCAGAGCATGA | GTAGCAAGAGGATGCCAGTGT |
|  | actin | AGGGTGTAGAGTGTTTGCAGTC | CTCAAGGTGGACAGATGCGG |

**Table 3.** siRNA sequences.

| **siRNA** | **Sequences (5’-3’)** |
| --- | --- |
| IDO1 siRNA#1-sense | GGGCUUUGCUCUACCACAUTT |
| IDO1 siRNA#1-antisense | AUGUGGUAGAGCAAAGCCCTT |
| IDO1 siRNA#2-sense | CCAUGACAUACGAGAACAUTT |
| IDO1 siRNA#2-antisense | AUGUUCUCGUAUGUCAUGGTT |
| IDO1 siRNA#3-sense | GCACUGCACGACAUAGCUATT |
| IDO1 siRNA#3-antisense | UAGCUAUGUCGUGCAGUGCTT |
| AhR siRNA#1-sense | CGCUGAAGGAAUUAAGUCATT |
| AhR siRNA#1-antisense | UGACUUAAUUCCUUCAGCGTT |
| AhR siRNA#2-sense | CUUCACACCUAUUGGUUGUTT |
| AhR siRNA#2-antisense | ACAACCAAUAGGUGUGAAGTT |
| AhR siRNA#3-sense | CAUCGACAUAACGGACGAATT |
| AhR siRNA#3-antisense | UUCGUCCGUUAUGUCGAUGTT |
| Control siRNA-sense | UUCCUCCGAACGUGUCACGUTT |
| Control siRNA-antisense | ACGUGACACGUUCGGAGAATT |

**Table 4.** The sequences of primers used for ChIP-qPCR analysis.

| **Primer** | **Sequences (5’-3’)** |
| --- | --- |
| DKK1-P1-forward | CCACCACATTATTCCACCAT |
| DKK1-P1-reverse | CAAGGCTAGTTACCCACAGA |
| DKK1-P2-forward | ATTTAAATATTGTTCATAATAT |
| DKK1-P2-reverse | ATCCTTGTTGGAGAGGGTGA |
| DKK1-P3-forward | TATAGGAGCCACCTGGGCTT |
| DKK1-P3-reverse | GAGCTTGAGTGTTCTGCTGT |
| DKK1-P4-forward | AAGGGGACCACAGTGCAAGG |
| DKK1-P4-reverse | CTCTGCACCGCCACCGCCAC |

**Materials and methods**

Cell culture

SD rat primary hippocampal neuron culture

Hippocampal neuron culture was prepared using method previously described by Banker et al.^1^ with minor modification. The hippocampi from embryonic SD rats were isolated and dissociated with trypsin for 15 min at 37 °C. The cell suspension was collected in a cell strainer to obtain the dissociated neurons that were then rapidly transferred into the medium. The neurons were then counted and plated on coverslips coated with poly-D-lysine in 12-well culture plates or 6-well culture plates and maintained in Neuro basal medium supplemented with B27 (Invitrogen, CA, USA). Half of the medium was replaced with an identical medium every 3 days. Cultures were kept at 37 °C in a humidified incubator with 5% CO_2_/95% air for 14 days before using.

HT22 culture

HT22 cells were cultured in Dulbecco’s Modified Eagle’s Medium (DMEM) (GIBCO, USA) supplemented with 10% (v/v) fetal bovine serum (FBS), 1% penicillin/streptomycin and NaHCO_3_ (2 mg/mL) and maintained at 37 °C in an atmosphere of 5% CO_2_ and 95% humidified air.

Compounds

RY101 and RY103 are the IDO1 inhibitors designed and developed by our lab. The chemical formulas of RY101 and RY103 are kept non-disclosed due to confidentiality reasons.

Western blot

Tissues or cells were lysed with RIPA lysis buffer. After centrifugation, the supernatants were collected as total proteins. Nuclear and cytoplasmic proteins were respectively extracted by the nuclear and cytoplasmic protein extraction kit (Beyotime, China) according to the manufacturer’s instructions. Protein concentration was determined with the BCA kit (Beyotime). Lysates (25-40 μg) were separated by 10% SDS-PAGE and transferred onto PVDF membranes. Proteins were probed by western blot assay. The resultant blots were visualized with ECL reagents (Thermo Fisher Scientific, USA) and immunoreactive signals were analyzed by densitometry using Image J software. The antibodies used were as follows: IDO1 (1:1000, Abcam, USA), AhR (1:1000, HuaBio, China), p-β-catenin (1:800, HuaBio), β-catenin (1:1000, Abcam), p-GSK3β (Ser 9) (1:1000; Beyotime), GSK3β (1:1000; Beyotime), p-Tau (T231) (1:1000, Beyotime), Tau (1:1000, Beyotime), GAPDH (1:2000, HuaBio).

RNA isolation and quantitative real-time PCR (qPCR)

Total RNA was isolated from the mice hippocampi or cells using TRIzol reagent (Takara, Japan). Reverse transcription was performed to synthesize cDNA using Premium One-Step RT-PCR kit (Invitrogen). qPCR was performed in triplicate for each sample using a SYBR Green Mastermix kit (Takara), actin was used as an internal control. The sequences of primers were shown in Table 2.

Immunofluorescence

The cells were fixed in 4% paraformaldehyde. Triton X-100 (0.2%) was used to permeabilize cells and blocking was done using with 10% normal goat serum (Beyotime C0265). The cells were incubated with primary antibodies overnight at 4°C, which was followed by the incubation with secondary antibodies for 1 h at room temperature. The following primary antibodies were used: IDO1 (1:100, Abcam), AhR (1:100, HuaBio), class III β-tubulin (1:200, HuaBio), PSD95 (1:200, HuaBio). The following secondary antibodies were used: Alexa Fluor 488 goat-anti-rabbit, Alexa Fluor 555 goat-anti-mouse. The cell nuclei were then stained with DAPI (1:1000). The cell slices were imaged using a laser scanning confocal microscope (Nikon Eclipse A1-Ni, Japan).

Flow cytometry

Cell apoptosis was analyzed by Annexin V-FITC/PI apoptosis detection kit (Yeasen Biotech, China). Cells were harvested and then washed twice with PBS. Cells were incubated with Annexin V-FITC and PI in 1× binding buffer for 15 min at room temperature in the dark. Annexin V-PI (AV-PI) double-stained cells were analyzed using a FACSCalibur flow cytometer (BD Biosciences, USA).

Animals

Pregnant SD rats were purchased from Shanghai Jiesijie Experiment Animal Co., Ltd. (Shanghai, China). 3-month-old male APOE^-/-^ (APOE knockout) mice, 3-, 6-, 9-, and 12-month-old male APP/PS1 (APPswe/PSEN1dE9 double-transgenic) mice and the corresponding WT mice (nontransgenic littermates of the APOE^-/-^ and APP/PS1 mice: WT^APOE-/-^ and WT^APP/PS1^) were obtained from the Model Animal Research Center of Nanjing University (Nanjing, China). The experimental procedures were approved by the Animal Ethics Committee of Fudan University and performed in compliance with ARRIVE guidelines.

Behavioral testing

Animals grouping and administration

8-month-old male APP/PS1 mice were randomly divided into four groups: control group, RY103 group, 1-L-MT group and donepezil group (8-15 animals per group). The RY103 group and 1-L-MT group were orally administered 50 mg/kg body weight of 1-L-MT or RY103 per 2 days. Donepezil group and control group were orally administered 1 mg/kg body weight of donepezil or an equal volume of 0.5% CMC-Na once daily. 1-L-MT, RY103 and donepezil were all dissolved in 0.5% CMC-Na. 8-month-old age-matched male C57BL/6 mice were selected randomly as WT group mice that were given 0.5% CMC-Na. All mice were orally administered for one month and then trained in the Morris water maze (MWM).

Morris water maze (MWM)

Mice were tested behaviorally after one-month feeding. The MWM test was carried out as previously described^2^.

Trypan Blue assay

All adherent and floating cells were harvested, and 10 µL of the combined cell suspension were incubated with 10 µL 0.4% trypan blue dye (Corning, USA) in PBS for 2 min in a 37 °C incubator to evaluate cell viability. Each cell suspension was mixed, pipetted into a hemocytometer (Invitrogen) and placed into a Countess cell counter (Invitrogen). Three readings (viable cell number and total cell number) were taken for each well, and all samples were run in triplicate. Percent viability was determined using the following equation: [1- (OD Expt-OD Blank) / (OD Air-OD Blank)] × 100%.

Transfection with siRNA

siRNA transfection was performed using Lipofectamine™2000 (Invitrogen) according to the manufacturer’s instructions. All siRNA oligos used in this study were purchased from Genepharma (Shanghai, China). The siRNA sequences were shown in Table 3.

Retroviral infection

Retroviruses carrying pBABE-mIDO1, pBABE-mAhR, pKLO-mIDO1, pKLO-mAhR vectors or empty vector were produced in HEK293T cells using VSVG and GAG as packaging plasmids. Retroviral supernatant was harvested 48  h after initial plasmid transfection and mixed with polybrene (8 μg/mL) to increase the infection efficiency. Stable cell pools were selected with 10 ng/L puromycin (Amresco, USA) for 5 days.

IDO1 activity assay

Blood samples were collected and centrifuged at 3000g for 15 min to obtain serum. The IDO1 activity in serum was evaluated by measuring the levels of Trp and Kyn with HPLC, as described previously^3^. Cell based IDO1 activity assay was performed according to reference^4^.

Chromatin immunoprecipitation (ChIP)-qPCR

Cells were crosslinked with 1% formaldehyde for 10 min at room temperature. The fixed cells were lysed and sonicated, the lysates were cleared by centrifugation, and antibody against AhR was added and incubated overnight under rotation^5^. After reversal of crosslink, precipitated DNA was purified with column and eluted in 20 μL water. Precipitated DNA fragments were amplified by qPCR. Primers specific for the DKK1 promoter were shown in Table 4.

Statistical analysis

All of the statistical analyses were conducted using Prism 6 software (GraphPad Software). One-way analysis of variance (ANOVA) followed by Dunnett’s post hoc test was used to compare several treatment groups with a control group. Student’s t-test was used to determine the difference between two independent groups. Significance values were set at **p* <0.05, ***p* <0.01 and ****p* <0.001.

**Acknowledgments**

This work was supported by the Key Biomedical Program of Shanghai (NO. 17431902200 & 18431902600) and Shanghai Municipal Science and Technology Major Project (NO. 2018SHZDZX01) and ZJLab.

Disclosures: The authors report no biomedical financial interests or potential conflicts of interest.

**Supplementary Data**

Supplementary data related to this article can be found at Supplemental Materials.

**REFERENCES**

1 Banker, G. A. & Cowan, W. M. Rat hippocampal neurons in dispersed cell culture. *Brain Res.* **126**, 397-342 (1977).

2 Vorhees, C. V. & Williams, M. T. Morris water maze: procedures for assessing spatial and related forms of learning and memory. *Nat Protoc.* **1**, 848-858 (2006).

3 Liang, H. *et al.* The proatherosclerotic function of indoleamine 2, 3-dioxygenase 1 in the developmental stage of atherosclerosis. *Signal Transduct Target Ther.* **4**, 23 (2019).

4 Yang, D. *et al.* N-Benzyl/Aryl substituted tryptanthrin as dual inhibitors of indoleamine 2,3-dioxygenase and tryptophan 2,3-dioxygenase. *J Med Chem.* **62**, 9161-9174 (2019).

5 Jordan-Pla, A. *et al.* SWI/SNF regulates half of its targets without the need of ATP-driven nucleosome remodeling by Brahma. *BMC Genomics.* **19**, 367 (2018).
